# Empowering hearts and shaping destinies: unveiling the profound influence of education on women’s mate selection in Pakistan – a comprehensive mixed-methods study

**DOI:** 10.3389/fsoc.2023.1273297

**Published:** 2023-12-21

**Authors:** Umar Daraz, Younas Khan, Ann Mousa Alnajdawi, Rula Odeh Alsawalqa

**Affiliations:** ^1^Department of Sociology, University of Malakand, Chakdara, Pakistan; ^2^Department of Sociology, Kohat University of Science and Technology, Kohat, Pakistan; ^3^Department of Rural Sociology, The University of Agriculture, Peshawar, Peshawar, Pakistan; ^4^Department of Social Work, The University of Jordan, Al Jubeiha, Jordan; ^5^Department of Sociology, The University of Jordan, Al Jubeiha, Jordan

**Keywords:** decision making, empowerment, women education, mate selection, mix-method, Pakistan

## Abstract

This study investigates the impact of education on women’s empowerment in the realm of mate selection within Malakand Division, Khyber Pakhtunkhwa, Pakistan. Employing a mixed-method research approach, the study conducted 30 semi-structured interviews with educated women and distributed a Likert Scale questionnaire to 500 women. The qualitative findings reveals that education significantly influences women’s perspectives, guiding their priorities, and instilling a desire for compatibility and shared values in their relationships. Educated women also display resilience, confidence, and a readiness to challenge prevailing societal norms and gender stereotypes when selecting a life partner. Quantitative analysis establishes a statistically significant positive correlation between education and women’s empowerment in mate selection. These findings underscore the pivotal role of education in enhancing women’s agency and autonomy in a critical aspect of their lives. The study’s implications extend to policy recommendations advocating for increased access to quality education and the implementation of gender-sensitive curricula in Malakand Division and analogous regions. Recognizing education’s potential to empower women in mate selection is vital for fostering a more equitable and inclusive society.

## 1 Introduction

In traditional societies like Pakistan, mate selection has historically been governed by cultural norms and familial expectations, often limiting women’s agency in choosing life partners. However, a significant transformation is evident with the rising levels of female education, granting them increased autonomy in marital decisions. Education serves as a powerful catalyst (Morris et al., 2006), enabling women to exercise choice in selecting a spouse. Existing literature e.g., Purewal and Hashmi (2015), Thomas (2023) highlights how education equips women to challenge traditional norms, allowing them to play a more active role in mate selection. Islamic teachings also underscore a woman’s right to consent in marriage, emphasizing her prerogative to choose a partner based on personal preferences (Aziz et al., 2022; Abdulmughni and Al-Abyadh, 2023; Ah-King, 2023). As women gain access to education, they acquire a heightened awareness of their rights, cultivating aspirations for equality and respect in their relationships. This newfound awareness motivates them to seek partners who share their values and envision a progressive and egalitarian partnership. Empirical research in this domain provides compelling evidence to support these assertions. Numerous studies demonstrate that as women attain higher levels of education, they demonstrate a stronger inclination toward seeking equitable and respectful relationships, reflected in their preference for partners who not only comprehend but actively endorse their aspirations for gender equality (Gangestad et al., 2006; Kim, 2023). Moreover, educated women are more likely to engage in open and constructive communication with potential partners. This proclivity for effective communication fosters a deeper understanding and enhanced compatibility in their relationships, positively impacting relationship satisfaction and longevity. This transition from passive conformity to cultural norms to active engagement in mate selection represents a significant and affirmative stride toward women’s empowerment in Pakistan, aligning with the global trend of women advocating for agency and autonomy in their personal lives, bolstered by education and awareness of their rights. These findings underscore the pivotal role of education in reshaping societal norms and advancing women’s empowerment within the context of mate selection (Aziz et al., 2021; Chan et al., 2023; Coates et al., 2023). Education emerges as a transformative force for women, with far-reaching impacts extending beyond mate selection to catalyzing broader societal transformations. Empirical evidence consistently affirms the pivotal role of educated women in challenging and reshaping detrimental practices, including early marriages and dowry demands, while concurrently advocating for gender equality. Educated women serve as influential role models, leaving an enduring imprint on future generations (Yasmin, 2013; Li and Choy, 2023). Research and empirical studies provide robust support for the idea that as women attain higher levels of education, they are more inclined to postpone marriage, a phenomenon observed in various regions, including Pakistan. This delay in marriage holds significant benefits, allowing women to pursue educational and career aspirations, contributing to their empowerment and overall well-being (Kreyenfeld, 2002; Klugman et al., 2014; Hamplová and Bičáková, 2022). Moreover, the health outcomes of educated women tend to be superior; they often enjoy enhanced access to healthcare services, make informed decisions about their reproductive health, and experience fewer instances of maternal mortality. This underscores the positive impact of education on women’s health and well-being. Education also plays a prominent role in societal productivity. Educated women are more likely to actively participate in the workforce, contribute to family income, and engage in community development activities. Their involvement is closely associated with improved socio-economic outcomes for their families and communities, underlining the multifaceted influence of education on women’s lives and society at large (Cornwall and Edwards, 2015; Nguyen-Phung and Nthenya, 2023; Tiwari and Malati, 2023).

In summary, education not only empowers women in mate selection but also instigates significant societal transformations. Supported by empirical evidence, education delays marriage, improves women’s health, and fosters social productivity. Educated women challenge detrimental practices and champion gender equality, leaving an enduring legacy as role models for future generations, advancing a more equitable and progressive society (Shetty and Hans, 2015; Hahn et al., 2018; Kim and Jung, 2023).

This study investigates the impact of education on women’s mate selection in Pakistan’s traditional society. Through comprehensive research and analysis, the aim is to examine how education influences women’s attitudes, preferences, and decision-making processes in the context of choosing a life partner. This exploration of women’s empowerment in mate selection contributes to the broader discourse on gender equality and social transformation in the country.

### 1.1 Problem statement

The process of mate selection in the Malakand Division, Khyber Pakhtunkhwa, Pakistan, has traditionally been shaped by long-standing customs and family-driven decisions, often relegating women to passive roles in choosing their life partners. However, the increasing prevalence of women’s education has introduced substantial changes, affording them the ability to make informed choices and assert independence in partner selection. Nevertheless, there remains a significant gap in understanding the impact of education on women’s mate selection in this region.

This study endeavors to delve into how education serves as a central catalyst in reshaping entrenched societal norms, empowering women with increased self-awareness and autonomy. Through an exhaustive examination of the connection between education and women’s empowerment in mate selection, our research aims to underscore the potential of education to challenge traditional practices and cultivate equitable partnerships grounded in mutual understanding and compatibility. Additionally, the study explores the influence of Islamic principles on women’s consent in marriage and how education reinforces this right, enabling women to articulate their preferences and needs.

Relying on an extensive body of research, including various studies and scholarly works, this investigation captures the lived experiences of women who have leveraged education to make empowered decisions concerning their marital choices. By shedding light on the impact of education on women’s mate selection in the Malakand Division, this research contributes to the broader discourse on gender equality and social transformation in the region. Ultimately, the findings of this study illuminate the effectiveness of educated choices in matters of the heart, paving the way for a more inclusive and equitable society in Khyber Pakhtunkhwa, Pakistan.

### 1.2 Situation of women in Malakand division

Malakand Division, like many other regions, has historically been influenced by deeply rooted customs and family decisions when it comes to mate selection. These customs often prioritize factors like family honor, social status, and economic considerations over individual preferences. Women, in particular, have had limited agency and autonomy in choosing their life partners. The introduction of education for women represents a significant departure from these traditional norms. As more women in Malakand Division gain access to education, they are exposed to new ideas, perspectives, and opportunities for personal development. Education provides them with valuable skills, knowledge, and self-awareness, empowering them to make informed decisions about their life partners. Women who receive an education often experience a newfound sense of self-worth and independence. Education equips them with the ability to critically evaluate potential partners based on compatibility, shared values, and personal preferences. This empowerment enables them to exercise their agency in mate selection and assert their rights to choose partners who align with their aspirations and goals. Education challenges traditional practices that might limit women’s choices and independence in mate selection. It fosters a shift toward equal partnerships where both parties have a say and contribute to decision-making, leading to healthier and more fulfilling relationships. In addition to education, this research also explores the influence of Islamic principles on women’s right to consent in marriage. It examines how education reinforces these principles, enabling women to assert their preferences and voice their needs within the framework of their religious beliefs.

The study aims to capture the real-life experiences of women who have benefited from education in making empowered decisions in their marital choices. These narratives will provide valuable insights into the practical impact of education on mate selection in the region. Ultimately, the research contributes to a broader discourse on gender equality and social change in Malakand Division and Khyber Pakhtunkhwa, Pakistan. It highlights the transformative potential of education in promoting inclusive and equitable societies, where both men and women have the opportunity to shape their futures, including their choices in matters of the heart.

In summary, education has the potential to empower women in Malakand Division by challenging traditional norms, fostering self-awareness and independence, and enabling them to make informed choices in mate selection. This research seeks to shed light on this transformation and its broader implications for gender equality and social change in the region.

### 1.3 Definitions of key concepts

#### 1.3.1 Education

Education is the process of acquiring knowledge and skills through structured learning, fostering personal and societal growth. It empowers individuals to understand the world and contribute to its betterment. Education is a central concept, emphasizing its role in empowering women and influencing their choices in mate selection.

#### 1.3.2 Mate selection

Mate selection is the process of choosing a partner for reproduction and companionship, influenced by factors such as attraction, compatibility, and evolutionary instincts. It plays a crucial role in shaping the genetic and social dynamics of a species. This concept highlights the core focus of the research, which is understanding how education shapes women’s perspectives and decisions in choosing life partners.

#### 1.3.3 Women empowerment

Empowerment is the process of granting individuals the tools, resources, and confidence to assert their rights, make decisions, and pursue their goals autonomously. Women empowerment specifically focuses on redressing historical gender disparities and enabling women to participate equally in social, economic, and political spheres, fostering gender equality. The study focuses on how education empowers women, giving them the agency to make informed decisions in their mate selection process.

#### 1.3.4 Malakand division

Malakand Division, a region in Pakistan, reflects entrenched gender norms, where women have traditionally had restricted decision-making power in mate selection, exemplifying significant gender inequality in this socio-cultural context.

### 1.4 Theoretical framework

The theoretical framework of this research paper is meticulously crafted to provide a solid foundation for understanding the impact of education on women’s empowerment in mate selection within the unique cultural context of Malakand Division, Pakistan. The theories and concepts employed are justified and logically linked to this specific region:

#### 1.4.1 Empowerment theory

In Malakand Division, education can empower women by equipping them with knowledge and critical thinking skills, enabling them to challenge traditional norms and advocate for their rights in mate selection. Given the region’s conservative cultural landscape, empowering women through education becomes even more crucial (Joseph, 2020).

#### 1.4.2 Gender and socialization theory

In this context, the influence of traditional gender norms on women’s roles and choices in mate selection is particularly pronounced. Education serves as a means to disrupt these norms, allowing women to express their preferences more freely in choosing life partners. This theory resonates with the socio-cultural dynamics of Malakand Division (Tabatadze and Gorgadze, 2023).

#### 1.4.3 Intersectionality theory

In a diverse socio-cultural context like Malakand Division, various factors, including gender, class, religion, and education, shape women’s experiences and opportunities. Understanding how education uniquely empowers women in this specific region is essential for a nuanced analysis (Merz et al., 2023).

#### 1.4.4 Incorporating Islamic principles on consent

The cultural and religious values of the predominantly Muslim population in Malakand Division align with the Islamic perspective on consent in mate selection. Education reinforces women’s right to consent, harmonizing with the local religious and cultural values (Showkat, 2023).

#### 1.4.5 Social change and gender equality perspective

Malakand Division, like many other regions in Pakistan, may grapple with harmful practices and gender inequality. Education’s role in challenging these practices and promoting gender equality contributes to progressive societal change. This perspective is particularly relevant for understanding the potential impact of education in Malakand Division (Edström et al., 2015).

In summary, this theoretical framework is not only logical but also highly justifiable in the context of Malakand Division, Pakistan. The unique socio-cultural dynamics of the region, with its blend of tradition, religion, and the need for progress, make education a pivotal factor in empowering women to make informed mate selection choices, challenge existing norms, and work toward a more inclusive and equitable society. It is through the lens of these theories that can comprehensively analyze data and better understand the profound significance of education in shaping women’s empowerment and marital decision-making within Malakand Division’s cultural context.

#### 1.4.6 Link of the theoretical framework with design and analysis of the study

The research design and data analysis for this study are intrinsically linked to the theoretical framework established earlier. The selected theories and concepts underpin the research design and guide the methodology. For instance, the Empowerment Theory informs the choice of exploring how education empowers women in mate selection. The Gender and Socialization Theory helps in crafting questions that address the disruption of traditional gender norms. The Intersectionality Theory ensures the diversity of the sample to account for various socio-cultural contexts. Incorporating Islamic Principles on Consent aligns with local cultural and religious values. The Social Change and Gender Equality Perspective guides the analysis to explore the impact of education in challenging harmful practices. Thus, the theoretical framework not only justifies the research design but also directs the analysis, resulting in a holistic understanding of women’s empowerment in mate selection within the unique context of Malakand Division, Pakistan.

## 2 Material and methods

This research paper utilizes an explorative sequential method (Creswell and Clark, 2017) to highlight the importance of education for women’s empowerment in mate selection in Malakand Division, Khyber Pakhtunkhwa, Pakistan.

### 2.1 Qualitative portion

#### 2.1.1 Universe population and target population

The universe population for this qualitative study comprises educated women in Malakand Division, Khyber Pakhtunkhwa, Pakistan. This region was selected due to its relevance to the research topic and its prevalence of educational opportunities for women. The target population is carefully selected and includes educated women from diverse backgrounds. This diversity encompasses teachers from schools, colleges, and even universities within the Malakand Division. The inclusion of this diverse group of women is well-justified because the issue at hand is directly related to educated women. The choice to focus on teachers within this age range (28–45) offers a solid justification within the universe population. These educators have a unique perspective on education’s role in women’s empowerment, making them a highly relevant and insightful subgroup within the broader universe population. Their experiences and insights can contribute significantly to understanding the impact of education on women’s mate selection in this region.

#### 2.1.2 Sampling and sample size

Purposive sampling is logically employed in the qualitative phase, adhering to the saturation point concept (Guest et al., 2020), which indicates that around 30 participants are carefully selected based on their educational backgrounds and willingness to share candidly. This method ensures that the sample represents the target population effectively. A sample size of 30 is justified due to the qualitative nature of the research, adherence to the saturation point concept, and the focus on education’s empowering impact on mate selection among women. This number ensures a rich and comprehensive exploration of participants’ perspectives while being mindful of resource limitations.

#### 2.1.3 Tools of data collection

Data collection is authorized by the University of Malakand, adhering to ethical guidelines. Semi-structured interviews are conducted, allowing participants to freely express their experiences. With explicit consent, the interviews are audio-recorded and transcribed verbatim for rigorous thematic analysis. This method ensures both ethical standards and a rich source of qualitative data for a comprehensive exploration of the empowering impact of education on mate selection among women.

#### 2.1.4 Data analysis

The utilization of thematic analysis, in accordance with Bryman’s method, is a logical choice for this study (Bryman, 2016). Thematic analysis is a well-established and robust qualitative research approach. It systematically examines interview transcripts, enabling the identification of recurring themes associated with women’s empowerment, decision-making autonomy, and the influence of education on mate selection. This approach ensures a methodical and rigorous examination of the data, aligning with the study’s aim to comprehensively explore the impact of education on women’s mate selection.

### 2.2 Quantitative portion

#### 2.2.1 Universe population and target population

The universe population remains the same- educated women in Malakand Division, Khyber Pakhtunkhwa, Pakistan. However, the target population for the quantitative portion differs from the qualitative portion, as it involves a larger and more diverse group of educated women.

#### 2.2.2 Sampling and sample size

Stratified random sampling is logically employed in the study due to the diverse strata of schools, colleges, and universities in the divorced region of Malakand Division. Using a disproportionate method, 500 female teachers aged 28–45 are selected from these strata. The sample size of 500 is justified as it provides adequate representation across diverse educational strata, enabling robust conclusions regarding the impact of education on mate selection among educated women in the Malakand Division. This larger sample size increases the study’s statistical power, allowing for more precise and reliable results, and accounts for potential variations in mate selection factors across different educational institutions.

#### 2.2.3 Tool of data collection

Employing structured survey questionnaires with a Likert scale allows systematic data collection, addressing factors such as educational attainment, decision-making autonomy, mate selection preferences, and the perceived role of education in marital choices. The inclusion of approximately 500 educated women in the target population ensures a comprehensive representation. Accessing respondents through educational offices for questionnaire delivery to teachers from various educational levels (schools, colleges, and universities) enhances diversity. This approach aligns with the study’s objective to provide broader insights and generalize findings on education’s influence on mate selection among women in the region.

#### 2.2.4 Data analysis

The utilization of SPSS for chi-square, regression, and correlation analyses to investigate the interplay between education and women’s empowerment in mate selection aligns with prior (Daraz et al., 2023) studies demonstrating that education can significantly influence women’s empowerment. Employing these statistical methods ensures a robust exploration of this connection, contributing to a growing body of empirical evidence.

### 2.3 Integration of qualitative and quantitative analysis

The integration of qualitative and quantitative data in an explorative sequential method is justified by the complexity of the research topic. Previous studies (Kågesten et al., 2016; Aziz et al., 2023) have demonstrated that education plays a multifaceted role in women’s mate selection in Pakistan. The inclusion of thematic analysis alongside quantitative data enhances the depth of analysis, aligning with the existing body of empirical evidence (Creswell and Clark, 2017).

### 2.4 Ethical considerations

Ethical considerations are of paramount importance in this study. The researchers prioritize the confidentiality and anonymity of all participants. Informed consent is obtained from each participant, ensuring they are fully aware of the study’s purpose and their rights. Participants have the right to withdraw from the study at any point without facing any adverse consequences. The research adheres strictly to ethical guidelines and protocols set by the relevant institutional review board, safeguarding the well-being and rights of all involved.

### 2.5 Limitations and overcoming of the limitations

The study has certain limitations, notably the reliance on self-reported data, which may introduce bias due to social desirability. To address this, the research emphasizes anonymity and confidentiality to encourage more honest responses. Another limitation pertains to potential underrepresentation of women from marginalized or hard-to-reach communities. Researchers counter this by ensuring a diverse sample and accounting for various socio-cultural contexts, thereby enhancing the study’s broader applicability.

## 3 Results

### 3.1 Qualitative results

The results of the thematic analysis offer a systematic and substantiated insight into how education empowers women in the context of mate selection within Malakand Division, Pakistan, based on the respondents’ arguments.

The analysis reveals that educated women prioritize their own happiness and personal fulfillment when choosing partners, aligning with the existing literature’s emphasis on education’s transformative influence. This transformation extends to broadening women’s perspectives and fostering a strong sense of self-fulfillment as a crucial criterion in their partner selection process. Education equips women with the confidence and discernment required for informed decision-making, highlighting the importance of self-esteem and self-efficacy. Moreover, it enhances their critical thinking abilities. The analysis aptly acknowledges that women’s choices are not made in isolation but are influenced by complex socio-economic factors and external pressures, reflecting the realistic complexity of mate selection dynamics.

The analysis underscores that educated women actively challenge cultural norms governing partner selection. They are notably inclined to seek partners who actively support their personal growth and ambitions, even when these aspirations conflict with traditional family expectations. This resonance with broader literature reflects education’s role as a catalyst for social change, with due consideration to the potential tensions it may introduce. The thematic analysis also highlights the role of autonomy in mate selection, as educated women seek partners who respect their independence and acknowledge the influence of education on their choices, in alignment with the articulated arguments of the respondents. The analysis acknowledges the potential concern of elitism, wherein educated women may predominantly seek partners with similar educational backgrounds, potentially perpetuating social inequalities. This valid observation underscores the socio-economic dimensions inherent in mate selection decisions.

In summary, the thematic analysis logically and effectively aligns with the respondents’ arguments and existing literature, elucidating how education empowers women to make assertive choices in partners based on their values and life goals. The analysis underscores the intricate interplay of socio-cultural factors and underscores the importance of a nuanced understanding of the role of education in the lives of women and its impact on mate selection within the specific context of Malakand Division, Pakistan (Tables 1–3).

**Table 1:** Cross tabulation of education and women empowerment in mate selection.

Indicators of women mate selection	Education		Total
	Agree	Disagree	
Education expanded women criteria of mate selection	491	09	500
Education *empowered women decision-making regarding mate selection*	493	07	500
Education increased agency and autonomy for women in mate selection	495	05	500
*Education breaking stereotypes and gender norms in mate selection*	498	02	500
*Education and women* greater financial independence in mate selection	390	10	500
The impact of education on women’s mate selection and emphasis on shared values and goals	488	12	500
Education women face societal pressures and choose partners of personal fulfillment	492	08	500
Education enable women to negotiate roles and responsibilities in relationships	489	11	500
Education empowering women to prioritize shared parenting preferences	498	02	500
Education empowering women to choose partners despite family resistance	496	04	500

Application of chi-square test			
Statistical tests	Value	Degree of freedom	Asymp.Sig.
Pearson chi-square	54.786	9	0.000
Likelihood ratio	12.564	9	0.000
Linear by linear association	10.657	1	0.000
*N* of valid cases	500		

**Table 2:** Regression.

Coefficients^a^				
Unstandardized coefficients		Standardized coefficients	*t*	Sig.
*B*	Std. Error	Beta		
0.333	0.021		15.593	0.000
0.667	0.021	0.816	31.465	0.000
a.Dependent variable: women empowerment in mate selection				

ANOVA^a^				
Sum of squares	Df	Mean square	*F*	Sig.
1.325	1	1.325	990.024	.000^b^
0.667	498	0.001		
1.992	499			
a.Dependent variable: women empowerment in mate selection				
b.Predictors: (constant), education				

Model summary				
Model	*R*	*R* square	Adjusted *R* square	Std. error of the estimate
1	0.816^a^	0.665	0.665	0.037
a.Predictors: (constant), education				

**Table 3:** Correlations of education and women empowerment in mate selection.

Correlation of education (IV) and women empowerment in mate selection (DV)		Dependent variable women empowerment in mate selection	Independent variable education
Women empowerment in mate selection (Dependent variable)	Pearson correlation	1	0.816^**^
	Sig. (2-tailed)		0.000
	*N*	500	500
Education (Independent variable)	Pearson correlation	0.816^**^	1
	Sig. (2-tailed)	0.000	
	*N*	500	500


**Correlation is highly significant at the 0.01 level (2-tailed).

#### 3.1.1 Results of chi-square test

The presented results offer a comprehensive and empirically substantiated perspective on how education empowers women in the realm of mate selection. Based on the responses of 500 participants, these findings shed light on the profound impact of education in redefining the criteria and decision-making process for women in Malakand Division, Pakistan.

A remarkable 98% of the respondents, equivalent to 491 out of 500, voiced their agreement that education expanded their criteria for choosing a life partner. Educated women, as evident from the data, prioritize qualities like intelligence, shared interests, and emotional intelligence over conventional factors such as socioeconomic status or physical appearance. This shift in preference signifies the transformational influence of education in broadening women’s perspectives and fostering a focus on qualities that contribute to meaningful and fulfilling relationships.

Furthermore, a substantial 493 out of 500 participants, almost 99%, acknowledged that education empowered them to make confident decisions in the mate selection process.

The data demonstrates that educated women exhibit a greater sense of agency and autonomy in choosing partners who align with their values and goals, illustrating their capacity to resist external pressures and make choices reflective of their aspirations.

The findings continue to reinforce the pivotal role of education, with nearly 99% of respondents (495 out of 500) expressing how education increases their agency and autonomy in mate selection. Education equips women with the knowledge and skills to make informed choices that prioritize personal fulfillment and aspirations, allowing them to exercise greater control in their partner selection.

The impact of education is further underscored by an overwhelming majority, as 498 out of 500 respondents recognized how education empowers them to challenge traditional gender roles and stereotypes. Educated women are more inclined to seek partners who support equality and mutual respect in their relationships, emphasizing the transformative role of education in reshaping gender dynamics.

Education’s influence extends to financial independence, as 78% of the respondents (390 out of 500) agreed that education led to greater financial autonomy, influencing their preference for partners who value their career aspirations and respect their financial independence. The compatibility of values and shared goals in mate selection is also deemed vital by nearly 98% of the respondents (488 out of 500).

Education equips women with the skills to negotiate roles and responsibilities within a relationship, as articulated by almost 98% of the respondents (489 out of 500). This implies that educated women seek more equitable and balanced partnerships, where decisions are made collaboratively, reflecting the strength of education in fostering more egalitarian relationships.

In terms of parenting preferences, 99% of the respondents (498 out of 500) recognized the role of education in prioritizing shared parenting values, as educated women seek partners who actively participate in raising children and share parenting responsibilities.

The empowerment resulting from education extends to facing societal pressures, with 492 out of 500 participants expressing how education empowered them to assertively choose partners despite potential family resistance. This underscores the prioritization of personal happiness and fulfillment in the mate selection process.

Lastly, the application of the chi-square test reveals a significant relationship between education and women’s empowerment indicators in mate selection, with a value of *p* of 0.000. This statistical significance underscores the pivotal role of education in influencing women’s mate selection preferences and the robustness of the findings.

In conclusion, the presented results offer a robust and well-justified account of how education empowers women in Malakand Division, Pakistan, in their mate selection. These findings underscore the transformative impact of education in expanding women’s criteria, enhancing their decision-making agency, challenging traditional norms, and fostering equality in relationships.

#### 3.1.2 Results of regression

The linear regression analysis reveals a significant relationship between education and women’s empowerment in mate selection. The standardized coefficient (Beta) for education is 0.816, indicating a strong positive association. This means that as the level of education increases, women’s empowerment in mate selection also increases significantly. The unstandardized coefficient (B) for education is 0.333, suggesting that for every unit increase in education, women’s empowerment in mate selection increases by 0.333 units. The *t*-value of 15.593 further supports the significance of this relationship (value of *p* = 0.000).

The ANOVA results indicate that the regression model is highly significant (value of *p* = 0.000), with an *F*-value of 990.024. This suggests that education as an independent variable significantly predicts women’s empowerment in mate selection. The *R*-squared value of 0.665 indicates that approximately 66.5% of the variance in women’s empowerment can be explained by education. This demonstrates a strong effect of education on women’s decision-making and preferences when choosing a life partner.

#### 3.1.3 Results of correlation:

The correlation analysis indicates a highly significant positive relationship between education and women’s empowerment in mate selection. The Pearson correlation coefficient between education (independent variable) and women’s empowerment in mate selection (dependent variable) is 0.816, with a value of *p* of 0.000. Similarly, the correlation coefficient between women’s empowerment in mate selection and education is also 0.816, with a value of *p* of 0.000. These results highlight a strong and positive association between education and women’s empowerment in mate selection.

## 4 Discussion

### 4.1 Qualitative discussion

Mate selection is a crucial life decision that profoundly impacts an individual’s happiness, well-being, and overall life trajectory. A comprehensive thematic analysis was conducted to explore how education influences women’s mate selection criteria and decision-making processes. This analysis focuses on the responses and narratives provided by women themselves, allowing us to understand the transformative effects of education from their perspective.

#### 4.1.1 Expanded mate selection criteria: the power of education

Through the narratives of our respondents, it becomes evident that education plays a significant role in expanding women’s mate selection criteria. Respondents emphasized how their educational journeys exposed them to diverse perspectives, broadened their horizons, and challenged their preconceived notions about what makes a suitable partner. One participant eloquently shared her view, saying,

“My education made me realize that there’s more to a partner than just financial stability. I started looking for qualities like intelligence, shared interests, and emotional compatibility. Education opened my mind to the idea of a partner who stimulates my intellect and shares my passions.”

These stories refers to the findings of research conducted by Juhn and McCue (2017), which emphasize that education encourages individuals to seek partners who share their intellectual interests and values, shifting away from more traditional factors like financial stability.

#### 4.1.2 Empowerment through informed decision-making

The respondents also highlighted how education empowers women to make more confident and informed decisions in the mate selection process. By providing them with critical thinking skills and the ability to recognize red flags in relationships, education equips women to navigate the complex world of dating and relationships. One respondent shared her experience, stating,

“Education taught me to identify warning signs in relationships. I’m now more cautious about red flags like controlling behavior or lack of respect. I will not settle for an unhealthy partnership.”

This theme aligns with the research of Khan (2021), which emphasizes how education promotes informed decision-making in mate selection. Khan’s work substantiates the idea that educated women are better equipped to make assertive and fulfilling choices when it comes to their partners.

#### 4.1.3 Challenging stereotypes and gender norms: a personal journey

Through the narratives, it becomes apparent that education empowers women to challenge traditional gender roles and stereotypes, encouraging them to seek partners who support equality and mutual respect. Respondents highlighted their determination to break free from restrictive stereotypes and redefine gender roles. One participant remarked,

“My education taught me that I do not have to conform to traditional gender roles. I want a partner who values my ambitions, respects my financial independence, and supports an equal partnership.”

This theme is consistent with the research conducted by Vikram (2023), which underlines the role of education in reshaping mate selection criteria. Vikram’s findings align with the respondents’ emphasis on challenging stereotypes and prioritizing equality and mutual respect in their partner selection.

#### 4.1.4 Financial independence: education as a catalyst

Another significant theme is the enhancement of financial independence through education. Respondents shared their personal experiences of how education opened doors to better career opportunities and financial stability. This empowerment influences their preference for partners who value their financial autonomy and ambitions. A respondent shared her journey, saying,

“Education helped me secure a better job and achieve financial independence. Now I seek partners who respect my career goals and financial autonomy.”

This theme aligns with the research conducted by Wang and Ou (2022), which corroborates the narratives of the respondents. Their research illustrates how education plays a pivotal role in enhancing women’s financial independence and, in turn, influencing their preferences in mate selection.

#### 4.1.5 Prioritizing shared values and goals in mate selection

The narratives of the respondents reveal that educated women often prioritize finding partners who share similar values, life goals, and aspirations. This underscores the importance of compatibility and mutual understanding in relationships. One participant shared her perspective, stating,

“My education made me realize that shared values and emotional compatibility are crucial. I want a partner who shares my life goals and values.”

This theme is well-supported by the insights from Sidhant and Bhawna (2023) research, which emphasize that educated women prioritize partners with shared values and emotional compatibility. Their findings support the importance of this theme in mate selection.

#### 4.1.6 Confronting societal pressures: a transformational journey

The narratives highlighted how education empowers women to confront societal pressures and make choices based on personal fulfillment rather than external influences. Respondents shared stories of resilience in the face of societal norms and expectations regarding marriage and partner choice. One respondent explained,

“My education gave me the confidence to make choices based on my personal fulfillment, even when faced with societal pressures. I refuse to settle for anything less.”

This theme aligns with the research of Coley et al. (2023), which emphasizes the role of education in empowering women to navigate societal pressures. The respondents’ stories underscore the transformative influence of education in enabling women to make assertive decisions based on personal happiness.

#### 4.1.7 Negotiating roles and responsibilities: equitable partnerships

Education equips women with the skills to negotiate roles and responsibilities within a relationship, ensuring a more equitable and balanced partnership. Educated women develop effective communication skills, challenge traditional gender norms, and seek partners who support their personal aspirations while promoting equality in decision-making. A respondent shared her experience, saying,

“Education taught me to communicate effectively and negotiate roles and responsibilities in my relationship. I want a partnership based on collaboration and mutual understanding.”

This theme aligns with the insights from (Wilson, 2015), which emphasize the role of education in promoting collaboration and negotiation of roles and responsibilities in relationships. Wilson’s research findings support the idea that education empowers women to negotiate roles and responsibilities within their relationships.

#### 4.1.8 Shared parenting preferences: building the future together

The narratives revealed that educated women prioritize partners who share similar views on parenting styles. This theme reflects the importance of open discussions and mutual agreement in raising children. A respondent emphasized,

“My education taught me the significance of shared parenting preferences. I want a partner who is aligned with me in building our family’s future together.”

This theme is supported by the research of Khoja-Moolji (2015), which highlights the influence of education on women’s parenting preferences in mate selection. The narratives of the respondents substantiate the importance of shared parenting preferences in their mate selection.

#### 4.1.9 Assertive mate selection despite family approval: the importance of autonomy

The narratives showcased that education can influence the level of importance given to family approval in mate selection. Educated women may be more assertive in their partner choices, even if they face resistance from family members. One respondent shared her story, explaining,

“My education taught me to prioritize my personal fulfillment. Even when faced with family resistance, I assertively choose my partner based on my own criteria.”

This theme is substantiated by the research of Mataji Amirroud et al. (2023), which focuses on the impact of education on women’s assertiveness in mate selection despite family approval. The narratives of the respondents strengthen the idea that education empowers women to prioritize personal fulfillment and assertive decision-making.

In conclusion, the narratives of the respondents provide invaluable insights into the transformative influence of education on women’s mate selection criteria and decision-making processes. Education empowers women to make informed, fulfilling, and equitable decisions in their mate selection process, promoting.

#### 4.1.10 Quantitative discussion

The results demonstrate that education plays a pivotal role in empowering women in their mate selection journey. Education expands their perspectives, enabling them to seek partners who share common values, life goals, and emotional compatibility. Educated women display a greater emphasis on personal growth, challenging traditional gender norms, and negotiating roles and responsibilities in relationships. This empowerment also equips women to confidently face societal pressures and make assertive choices, even in the face of family resistance.

Education fosters a mindset that values compatibility and shared values in relationships. Educated women prioritize emotional intelligence, open communication, and collaborative decision-making, creating more fulfilling and equitable partnerships. Moreover, education provides women with increased financial independence, leading them to seek partners who support their career aspirations and respect their autonomy.

In conclusion, education empowers women to make informed and fulfilling choices in selecting a life partner. It breaks traditional stereotypes and enables women to assert their preferences confidently. The impact of education on mate selection is evident through the emphasis on shared values, emotional intelligence, and mutual respect, leading to healthier and more successful relationships. Education is a transformative force that promotes self-discovery, critical thinking, and a pursuit of happiness in women’s mate selection journey.

The chi-square test results revealed a significant and robust relationship between education and women’s empowerment indicators in the context of mate selection. With a value of *p* of 0.000, the findings highlight the crucial role of education in shaping women’s preferences and choices when it comes to selecting a life partner. Educated women demonstrate a preference for qualities like intelligence, emotional intelligence, shared interests, and compatibility over traditional factors like socioeconomic status or appearance. They display increased confidence and agency in decision-making, prioritizing personal fulfillment and aspirations over external pressures and societal norms.

Furthermore, education empowers women to challenge and break free from restrictive gender stereotypes and roles, seeking partners who embrace equality and mutual respect in relationships. It fosters effective communication and negotiation skills, allowing women to actively engage in decision-making within a partnership. Additionally, education influences women’s perspective on parenting, leading them to seek partners who share similar parenting preferences, promoting a more collaborative and supportive parenting dynamic.

In a nutshell, the chi-square test underscores the transformative power of education in empowering women during the mate selection process. As women gain knowledge and confidence through education, they are better equipped to make informed and fulfilling choices in selecting a life partner. Education emerges as a key factor in challenging traditional norms, prioritizing shared values and emotional compatibility, and fostering more egalitarian and successful relationships.

The quantitative discussion aligns with a body of previous empirical evidence that underscores the transformative impact of education on women’s mate selection choices. Studies such as those by Gautam and Jeong (2019) and Dalal (2011) have also found a significant relationship between education and women’s empowerment in the context of mate selection. These findings reinforce the consistent pattern that educated women prioritize shared values, emotional intelligence, and compatibility over traditional factors like socioeconomic status or appearance.

The linear regression analysis provides compelling evidence of the positive impact of education on women’s empowerment in mate selection. As women’s education level increases, they demonstrate higher levels of empowerment in making decisions related to choosing a life partner. This aligns with previous research indicating that education plays a crucial role in expanding women’s perspectives and empowering them to prioritize qualities such as shared values, emotional intelligence, and compatibility over traditional factors like socioeconomic status or appearance.

The significant standardized coefficient (Beta) of 0.816 underscores the importance of education in shaping women’s preferences and choices during mate selection. Educated women are more likely to assertively choose partners based on personal fulfillment, aspirations, and shared values rather than succumbing to societal pressures or family expectations.

The strong R-squared value of 0.665 signifies that education explains a substantial portion of the variance in women’s empowerment in mate selection. This highlights the transformative role of education in breaking stereotypes, challenging gender norms, and promoting more egalitarian and fulfilling partnerships.

The linear regression analysis aligns with studies by Naz and Ashraf (2020) and Kim (2023), which emphasize that as women’s education levels increase, they exhibit a higher degree of empowerment in making mate selection decisions. The strong standardized coefficient (Beta) and R-squared value mirror the consistent findings in these prior studies.

The correlation analysis provides robust evidence of the positive impact of education on women’s empowerment in mate selection. The significant correlation coefficient of 0.816 indicates that as the level of education increases, women’s empowerment in mate selection also increases. This suggests that education plays a vital role in empowering women to make informed and fulfilling choices when it comes to selecting a life partner.

The highly significant value of *p* of 0.000 further emphasizes the strength of the correlation between education and women’s empowerment in mate selection. This means that the observed relationship is unlikely to be due to chance and is indeed a meaningful and reliable association. The strong correlation between education and women’s empowerment in mate selection supports the previous findings from the linear regression analysis. Educated women are more likely to prioritize shared values, emotional intelligence, and compatibility over superficial factors when choosing a life partner. Education equips women with knowledge, critical thinking skills, and self-confidence, allowing them to navigate societal pressures, challenge stereotypes, and assertively make decisions that align with their personal fulfillment and aspirations.

Moreover, the correlation analysis in of the study refers to the results of research by Batool (2018) and Eastwick et al. (2006), which similarly highlight the positive correlation between education and women’s empowerment in mate selection. The robust correlation coefficient and significant value of *p* substantiate the reliability of this relationship.

In conclusion, the correlation analysis affirms that education is a crucial determinant of women’s empowerment in mate selection. As women’s education level increases, they display higher levels of empowerment in making decisions related to their life partner. The results highlight the transformative role of education in promoting women’s agency and autonomy, leading to more fulfilling and successful relationships based on mutual understanding and shared values.

## 5 Conclusion

In the context of the exploratory research conducted in Malakand division, Pakistan, focusing on the empowerment of women in mate selection through education, the comprehensive analysis, both qualitative and quantitative, yields a compelling and consistent conclusion. Education indeed emerges as a transformative force, empowering women to make informed and fulfilling choices in their pursuit of life partners.

Through qualitative analysis, it becomes evident that education broadens women’s perspectives, instills resilience and confidence, and empowers them to challenge societal norms and gender stereotypes. Women empowered by education prioritize compatibility, shared values, and personal fulfillment over external influences. They confidently negotiate roles in relationships, seek collaborative partnerships, and value open communication, ultimately fostering environments of love and support.

Quantitative analysis reinforces these qualitative findings, demonstrating a strong positive correlation between education and women’s empowerment in mate selection. Educated women exhibit expanded criteria, agency in their decisions, and a preference for partners who embrace equality and mutual respect. Education significantly predicts women’s empowerment in mate selection, explaining a substantial portion of the variance in their preferences.

In summary, this research underscores that education plays a pivotal role in empowering women in Malakand division, Pakistan, during their mate selection journey. It equips them with knowledge, critical thinking skills, and self-assurance, enabling them to challenge societal pressures, break stereotypes, and confidently choose partners based on shared values and personal growth. The transformative power of education is not only evident in mate selection but also holds broader implications for gender equality and overall well-being in society.

These findings emphasize the importance of continued efforts to promote and invest in women’s education, ultimately paving the way for a more equitable society where women are empowered to shape their lives and relationships with autonomy, confidence, and mutual respect.

### 5.1 Integration of the qualitative and quantitative data (triangulation)

The explorative sequential method is a research approach that combines qualitative and quantitative data collection and analysis to comprehensively understand a phenomenon. In the context of studying how education empowers women in mate selection in Malakand Division, Pakistan, this method provided valuable insights.

Qualitative data, gathered through thematic analysis, illuminated the profound impact of education on women’s mate selection. Education broadened their perspectives, reshaped priorities, and encouraged a preference for compatibility and shared values in relationships. Educated women demonstrated resilience and confidence, challenging societal norms and seeking supportive partners.

Quantitative analysis reinforced these findings, showing a significant positive correlation between education and women’s empowerment in mate selection. Increased education levels led to higher empowerment. Education explained a substantial portion of variations in mate selection preferences, underlining its importance.

This integrated approach highlights education’s transformative role. It equips women with knowledge and confidence, enabling them to navigate societal pressures and make assertive choices based on personal fulfillment and shared values. Education fosters emotional intelligence, communication skills, and negotiation abilities, resulting in more successful and equal relationships.

The research conducted in Malakand Division, Pakistan, on the empowerment of women in mate selection through education has significant theoretical and practical implications:

#### 5.1.1 Theoretical implications

The research in Malakand Division, Pakistan, underscores the significance of education in shaping women’s choices in mate selection. It aligns with established theories such as Social Learning Theory and empowerment theories, highlighting how education empowers women to challenge societal norms, prioritize compatibility, and seek partners who value equality. Additionally, it emphasizes the importance of recognizing the intersectionality of gender and education in understanding these dynamics, contributing to the field of relationship science. This research enhances our theoretical understanding of how education can be a transformative force in women’s mate selection, shedding light on the complex interplay between education and societal norms.

#### 5.1.2 Practical implications

The practical implications of this research call for concrete actions and policies. Firstly, governments and policymakers should prioritize investments in women’s education, recognizing its transformative power. Comprehensive gender equality policies are crucial, addressing discrimination in education, employment, and society. Women’s access to family planning and reproductive rights should be ensured to grant them autonomy in life and relationship planning. Supportive work-life policies, such as parental leave and flexible working arrangements, enable women to pursue careers and find partners who support their aspirations, contributing to healthier and more fulfilling partnerships. Educational programs that promote gender equality, critical thinking, and emotional intelligence should be developed. Additionally, awareness and advocacy efforts are essential to highlight the importance of women’s education and advocate for gender equality in society. This research offers practical guidance for creating a more equitable and empowering environment for women.

### 5.2 Recommendations

Governments should enact and enforce comprehensive gender equality policies, spanning education, employment, and family life, with the aim of eradicating discrimination. Anti-discrimination measures must be introduced to combat biases in both workplaces and society. Ensuring women’s access to family planning and reproductive rights is essential, granting them autonomy in life and relationship planning. The implementation of supportive work-life policies, including parental leave and flexible working arrangements, will enable women to pursue careers and find partners who support their professional aspirations, ultimately contributing to healthier and more fulfilling partnerships.

### 5.3 Limitations and gap for future researchers

This study carries inherent limitations that should be considered, particularly in the context of gender norms in Pakistan. Its focus on the Malakand Division of Pakistan may limit the generalizability of findings to other regions with varying cultural norms and societal expectations. Pakistan is a diverse country with distinct regional and cultural variations, and gender norms can significantly differ across these regions. As such, the impact of education on women’s mate selection may vary considerably. Furthermore, the reliance on self-reported data introduces the possibility of social desirability bias, which can be particularly relevant in a conservative cultural setting like Pakistan, where adhering to societal norms and expectations is often prioritized. This may influence the accuracy of the results, especially concerning sensitive topics like mate selection. The study’s omission of confounding variables, such as economic status, urban–rural disparities, and family structures, is another limitation. In Pakistan, gender norms are intertwined with economic disparities and varying family dynamics, which can significantly shape women’s mate selection preferences. The cross-sectional design provides a snapshot of women’s preferences at a particular moment, but a longitudinal approach would be more informative in understanding how education’s effects on mate selection evolve over time in response to changing gender norms.

Despite these limitations, the study contributes valuable insights into the complex interplay between education and women’s empowerment in mate selection within the specific context of the Malakand Division, Pakistan. Future research should consider these gender norms and regional variations, adopting more comprehensive methodologies to explore the multifaceted dynamics of education and mate selection in this context. Additionally, investigations into the role of digitalization, social media, and peer networks are crucial for understanding how education empowers women to challenge traditional gender norms and make informed choices in a changing Pakistani society.

## Data availability statement

The raw data supporting the conclusions of this article will be made available by the authors, without undue reservation.

## Ethics statement

The studies involving humans were approved by University of Malakand Chakddara Ethical Review Board. The studies were conducted in accordance with the local legislation and institutional requirements. The participants provided their written informed consent to participate in this study.

## Author contributions

UD: Conceptualization, Data curation, Formal analysis, Investigation, Methodology, Resources, Software, Supervision, Writing – original draft. YK: Methodology, Visualization, Writing – review & editing, Funding acquisition, Project administration. AA: Conceptualization, Funding acquisition, Methodology, Writing – review & editing. RA: Conceptualization, Funding acquisition, Methodology, Writing – review & editing.
